# Early warning scores: breaking or building barriers to critical care

**DOI:** 10.1186/cc13234

**Published:** 2014-03-17

**Authors:** E Dunne, RO Leary, K Srinivasan, B Ahmed, D Galvin, R Ni Mhuircheartaigh, B Marsh

**Affiliations:** 1 Mater Misericordiae University Hospital, Dublin, Ireland; 2 Tallaght Hospital, Dublin, Ireland; 3 St Vincent's University Hospital; 4 University College Hospital, Galway, Ireland

## Introduction

A prospective multicentre observational study was carried out to assess the extent to which critical care teams manage patients in hospital who are cared for outside the critical care unit. The Health Service Executive (HSE) in Ireland is in the process of implementing a national Early Warning Score (EWS) and, at an EWS of 7 or above, a referral to critical care is recommended. This study recorded the EWS of patients referred to the critical care team and describes the subsequent interventions made by the critical care team and patient outcomes.

## Methods

Six critical care departments in university-affiliated hospitals across Ireland collected data on all referrals to the critical care team over a 6-week period. Data were anonymised, coded and analysed centrally.

## Results

A cumulative total of 399 calls were made to the critical care teams in the six hospitals. The most common reason for referral was to request a critical care review of a patient (*n *= 319, 79.9%). Other reasons for referral included cardiac arrest, request to transfer patients from other hospitals and requests for vascular access. The average duration spent by the critical care team reviewing patients on the wards was 57 minutes. This increased up to 67 minutes for cardiac arrest calls. Of the 319 critical care reviews, 160 (50.2%) patients were subsequently admitted to critical care. A total 118 of this 160 had EWS of 7 or above, while 42 scored less than 7 but were still deemed to require admission to critical care.

## Conclusion

Regardless of the EWS, critical care teams are heavily involved in the management of patients outside critical care units. Fifty per cent of patients reviewed by the critical care team subsequently required admission to a critical care unit. The trigger threshold (7 and above) for referral to a critical care team currently recommended by the EWS escalation protocol is more likely to predict need for critical care admission. However, one in four patients referred below the threshold also required admission to a critical care environment. This study questions the safety of introducing such a protocol into acute hospitals. Will noncritical care staff be forced to wait until patients deteriorate further and reach the trigger threshold for referral or will the role of the critical care team expand further to look after all patients with abnormal EWS in hospital?

